# Spatialisation of Electricity Consumption in China Based on Nighttime Light Remote Sensing from 2012 to 2023

**DOI:** 10.3390/s25071963

**Published:** 2025-03-21

**Authors:** Yanshu Wang, Mingquan Wu, Zheng Niu

**Affiliations:** 1 International Research Center of Big Data for Sustainable Development Goals, State Key Laboratory of Remote Sensing Science, Aerospace Information Research Institute, Chinese Academy of Sciences, Beijing 100094, China; wangyanshu24@mails.ucas.ac.cn (Y.W.); niuzheng@radi.ac.cn (Z.N.); 2 University of Chinese Academy of Sciences, Beijing 100049, China

**Keywords:** China, NPP-VIIRS, nighttime light data, spatialisation of electricity consumption, time–space variation

## Abstract

The collection of spatialised electricity consumption data is considered of crucial importance for planning electric power facilities and achieving the United Nations Sustainable Development Goal 7 (SDG7). However, the predominance of statistical data on electricity consumption in China in combination with the lack of spatialised electricity consumption data for the past five years poses a serious challenge. To effectively address this issue, a nighttime light remote sensing estimation model of China’s electricity consumption was developed in this work. Specifically, NPP-VIIRS nighttime light and publicly available electricity consumption data were used, and a spatialised Chinese electricity consumption data product for the period 2012–2023 was derived. At the same time, the time–space variation of China’s electricity consumption was systematically analysed. For the spatial dimension, the power function model was proven to be the most suitable estimation model for China, with an average R^2^ of 0.9385, while for the temporal dimension, the quadratic polynomial model was the most suitable, with an R^2^ of 0.9706. From the analysis of time–space variation, an increase in both the number and extent of high electricity consumption areas was observed, particularly in third- and fourth-tier cities in the south, while some industrial cities experienced a decline in electricity consumption.

## 1. Introduction

Since energy is undoubtedly the driving force of economic and social development, electricity is one of the main pillars of the modern energy system [1,2]. To promote global access to electricity, the United Nations 2030 Agenda for Sustainable Development has adopted the SDG7.1.1 electrification rate as the first indicator of SDG7 (Affordable Clean Energy). The accurate and timely monitoring of data to assess the degree of electrification is considered the basis for the planning and construction of electrical infrastructure [3], which is crucial for achieving the SDG7.1.1 access rate target. At the same time, the advancement of renewable energy sources like wind and solar power is placing escalating demands on the capacity of grid power management. To this end, the collection of spatialised electricity consumption data is not only important to accurately monitor the degree of electrification in different regions but also plays a key role in the planning and construction of new energy power plants, such as wind power and solar energy plants. As a result, the level of grid power dispatch can be significantly improved which will, in turn, help achieve the goals of global power accessibility and energy transition.

However, the currently available data on electricity consumption in China are mainly statistical data of administrative units at various levels. These data are non-spatialised and cannot fully reveal the spatial distribution of electricity consumption. The development of a spatialisation method for electricity consumption by utilising nighttime light remote sensing imagery is regarded as a vital technical approach to address this issue [4,5]. Nighttime light remote sensing originated from the Operational Linescan System (OLS) of the U.S. Defence Meteorological Satellite Program (DMSP) [6,7,8]. Its successor, the Suomi National Polar-Orbiting Partnership (Suomi NPP) satellite with the Visible Infrared Imaging Radiometer Suite (VIIRS) on board, is currently used [9,10]. With the development of technology, different countries and organisations have launched nighttime light remote sensing satellites, such as the International Space Station (ISS) [11], Argentina’s SAC-C satellite [12], Israel’s EROS-B satellite, and China’s Jilin I 03 satellite, Luo Jia I 01 satellite [13], and SDGSAT-1 satellite. At present, DMSP-OLS and NPP-VIIRS nighttime light data are still the main data sources for nighttime light remote sensing research. These sensors provide the possibility of quickly obtaining the spatial and temporal dynamic patterns of regional power consumption and provide good data support for the spatialisation of power consumption.

Research on electricity consumption through the use of nighttime light remote sensing imagery has significantly advanced over the past few years. This area of study initially focused on confirming the feasibility of using nighttime light data for estimating electricity consumption [14,15,16] and exploring the correlations between the nighttime light index and electricity consumption across various spatial scales [17]. However, the research has since progressed to encompass quantitative modelling, spatial analysis and the examination of spatial–temporal dynamics related to electricity consumption. He et al. [18] combined the saturation correction of DMSP-OLS data with the normalised difference vegetation index (NDVI) to establish a relationship model between the provincial electric power consumption (EPC) and total nighttime light (TNL). The authors simulated the spatial grid data of China’s electricity consumption from 2000 to 2008, confirming the reliability of using DMSP-OLS data to fit the EPC in mainland China. Shi et al. [19] calibrated the global DMSP-OLS data from 1992 to 2013 using the invariant target region method, constructed a global EPC estimation model, and analysed the spatial and temporal dynamics of global EPC. Their methodology represented a significant step in understanding how electricity consumption patterns change over time. Townsend et al. [20] added an overglow removal model (ORM) to the base model of the state’s electric power consumption and DMSP-OLS nighttime light data and estimated a more accurate EPC at a higher spatial resolution. The authors highlighted the importance of refining modelling techniques to improve data accuracy. In 2012, after the launch of the NPP-VIIRS sensor, Shi et al. [21] used the NPP-VIIRS nighttime light data to establish a correlation between the TNL and EPC for each province and prefecture in mainland China. In parallel, the electricity consumption of Chinese provinces and cities in 2012 was regressed, demonstrating that NPP-VIIRS data outperformed DMSP-OLS in terms of accuracy when simulating electricity consumption at the provincial level. Zhang Li et al. [22] calibrated DMSP-OLS and NPP-VIIRS nighttime light remote sensing images and formed the DMSP-OLS and NPP-VIIRS Chinese city nighttime light datasets for 1992–2013 and 2013–2018. These datasets were next fitted to the electricity consumption in spatial dimensions. It was found that compared to DMSP-OLS, NPP-VIIRS nighttime light images were the most ideal data sources for EPC simulation. Falchetta et al. [23] used the inter-annual variation of NPP-VIIRS nighttime light remote sensing imagery from 2012 to 2016 to predict the EPC changes in different countries around the world and considered the relationships between the prediction effect and the income level and region. The results showed that the prediction accuracy was more stable in low- and middle-income countries and the prediction effect of the model was different in different regions, exhibiting certain relationships with local regional characteristics.

While studies to date have investigated the use of nighttime light remote sensing for estimating electricity consumption across different scales—global, provincial, prefectural, and county—encompassing both spatial and temporal dimensions, there is a notable paucity of research focused on predicting and spatialising electricity consumption at the national level in China. Moreover, there is a lack of comparative studies on the nighttime light estimation models of electricity consumption under different methods. Additionally, the latest electricity consumption estimation data have not been updated since 2018, with the current data unable to adequately meet the current demands of China’s power development and related sectors.

To effectively address these problems, a method to spatialise China’s electricity consumption from 2012 to 2023 based on NPP-VIIRS nighttime lighting data was proposed. The main objectives of this study were: (1) to construct a model of China’s electricity consumption at multiple spatial scales, long and short time series, and in both spatial and temporal dimensions, from which the optimal fitting model can be selected; (2) to produce a product of China’s spatialised electricity consumption data for the last 12 years; and (3) to analyse the characteristics of China’s time–space variation in electricity consumption. Our work provides valuable data support for the planning and allocation of China’s electric power facilities, which can be used to realise the SDG7.1 energy supply strategy in China.

## 2. Study Area and Data

### 2.1. Study Area

China is located in the eastern part of Asia, on the west coast of the Pacific Ocean. Over the last 12 years, China’s electricity consumption has been growing at a fast pace, with electricity consumption in 2023 being nearly twice as high as in 2012. Due to the constant increase in the demand for electricity, China’s total electricity production showed a continuous growth trend from 2012 to 2023. After the restructuring of energy sources, the share of renewable energy sources, such as hydropower, wind power, solar power, and nuclear power, of the total electricity production in China has gradually increased. Nonetheless, coal remains the main source of electricity production in China. To meet the growing demand for electricity and the need to optimise its energy structure, China has continued to expand its installed capacity of electric power generation during the 2012–2023 period by building new power plants, expanding existing power plants, and introducing advanced power generation technologies. At the same time, it has actively promoted the development of clean energy sources, experiencing substantial growth in the installed capacity of wind and solar energy. Investments in construction have also increased and power grids have been upgraded. Moreover, the construction of smart grids has been gradually promoted, and advanced information and communication technology have been utilised to achieve intelligent management and control of the power system. China has also continued to make efforts to maintain a balance between the supply and demand of electricity and achieve SDG 7.1.

### 2.2. Data and Data Preprocessing

#### 2.2.1. China Electricity Consumption Data

Data on the electricity consumption of administrative units at all levels in China from 2012 to 2023 were obtained. The data were mainly sourced from the National Energy Administration of China (https://www.nea.gov.cn/, accessed on 25 August 2024), the statistical yearbooks of the National Bureau of Statistics (https://www.stats.gov.cn/, accessed on 17 July 2024), and the provincial and prefectural statistical bureaus (for example, the website of the Beijing Municipal Bureau of Statistics, https://tjj.beijing.gov.cn/, accessed on 20 October 2024), including annual, quarterly, and monthly data. Due to the lack of electricity consumption data for Taiwan, Hong Kong, Macao, and Inner Mongolia, the study area did not include these regions. The electricity consumption data for January and February were unavailable for certain regions and were, therefore, excluded from this work.

#### 2.2.2. NPP-VIIRS Nighttime Light Data

In this study, annual 2012–2023 and monthly January–December 2020 NPP-VIIRS nighttime light data were provided by NOAA. These data can be accessed at the following link: https://eogdata.mines.edu/products/vnl/, accessed on 27 December 2024. Compared with DMSP OLS data, the NPP-VIIRS nighttime light data have superior temporal, spatial, and radiometric resolution [9], remain unsaturated, and can be radiometrically calibrated on-orbit to make the data comparable between the different time periods. Because of the high sensitivity, the detector can easily capture various bright sources, such as fires, volcanoes, auroras, etc. Although the data product has removed some clouds and stray light, there is still a certain amount of background noise and outliers which need to be denoised to minimise the impact of noise [21].

In this work, the increase in light duration and light intensity in summer and cloud cover resulted in a serious lack of data for the Chinese region in May, June, and July 2020. For this reason, the nighttime light data for May, June, and July were excluded from the study of monthly data. In the time regression of the monthly data, the sample points used were March, April, and August–December, totalling seven months.

#### 2.2.3. Data Preprocessing

The preprocessing of NPP-VIIRS nighttime light data mainly included cropping, reprojection, denoising, and nighttime light index calculation.

The nighttime light data from 2012–2023, and March, April, and August–December 2020, were cropped using the boundaries of China. Next, the images were reprojected to the WGS84 geographic coordinate system and the Albers conic equal area projection coordinate system to ensure minimum area distortion. Finally, the images were resampled to a spatial resolution of 500 m. Twelve annual and seven monthly datasets of China’s regional nighttime light images were obtained.

The denoising of NPP-VIIRS nighttime light remote sensing images primarily involved background noise and outlier processing, including pixel negative and extreme values. At present, there are two main processing methods for noise: one is to use DMSP-OLS nighttime light images as a mask to reject unstable light sources on NPP-VIIRS nighttime light images [24]. Because the DMSP-OLS data do not have background noise, these data can have some attenuation effects on the noise level. However, this method ignores newly added or absent lights in a short period of time while extracting the effective lights. On top of that, the negative values are not processed, which is not appropriate for noise removal in long time series and rapidly developing areas. The other method involves the consideration of large water areas, such as lakes and reservoirs, as the unlit areas, the establishment of random points in these areas, and the calculation of the average luminance values (Digital Number) of the random points. Thus, the low threshold of light brightness can be derived; an image Digital Number below the low threshold is assigned a value of 0 [25]. At the same time, it is believed that the maximum lighting value of a region should not exceed that of the most developed city in the region. Thus, the highest lighting value in the centre of the city was selected as the maximum lighting threshold, and the DN values higher than the maximum lighting value were assigned as the maximum lighting threshold. This method effectively eliminates negative values in the image and helps reduce noise caused by extreme values. However, since the high threshold for removing extreme values is determined based on high-energy-consuming cities, some extreme values may persist in low-energy-consuming cities after denoising. As a result, the removal of extreme values in these areas is less effective, and the residual noise can affect the model fitting accuracy in these regions. A method of filtering background values and abnormal noise by combining median filtering and low threshold denoising has also been examined in the literature [26]. In this work, the GDP of Ganzhou City was estimated with the data processed by this method, and the results showed that the estimation accuracy of the model established by this method was higher. Nevertheless, the denoising effect of this method applied to a large area has not yet been verified. Therefore, the thresholding method was used to remove noise from the images, considering the specific characteristics of China. The specific steps were as follows:

(1) Generate random points within an extensive water body area (e.g., Qinghai Lake, Poyang Lake), compute the average luminance value (DN) to establish the lower threshold, and zero out pixels with DN values below the lower threshold.

(2) Determine the maximum luminance value of high-energy-consuming cities (e.g., Beijing, Shanghai) through statistical analysis to set the upper threshold, which is assigned to pixels exceeding the upper threshold.

Finally, the denoised NPP-VIIRS nighttime light data were used to calculate various indices, such as the TNL, and the year-by-year total nighttime light values at the national, provincial and prefectural levels were obtained from 2012 to 2023. Additionally, the monthly total nighttime light values for March, April, and August–December 2020 were obtained at the national, provincial, and prefectural levels.

## 3. Methods

**Figure 1:**
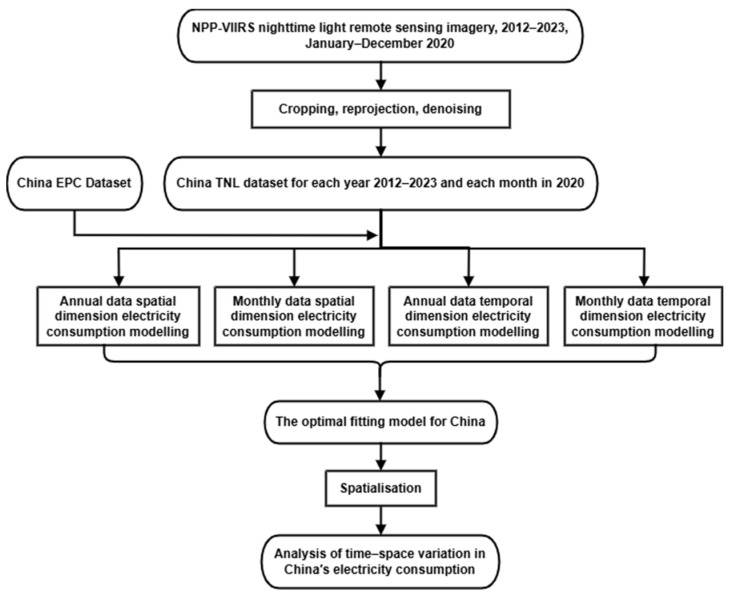
Flowchart of the research process.

In this work, the NPP-VIIRS nighttime light remote sensing images of March, April, and August–December 2020 as well as 2012–2023 were cropped, reprojected, and denoised. Next, the nighttime light index was calculated to obtain the total nighttime light dataset. Then, using the Chinese electricity consumption dataset and the total nighttime light dataset in the spatial and temporal dimensions, the TNL–EPC model was constructed and the best-fitting model was selected. The optimal fitting model was used to produce a spatial distribution map of China’s electricity consumption over the past 12 years and analyse the time–space variation in electricity consumption. The specific flow of the study is shown in Figure 1.

### 3.1. Modelling of TNL–EPC

The commonly used models for estimating electricity consumption include the linear, exponential, logarithmic, power function, and polynomial models. Table 1 presents details of the general form of the five models used in this work. Using these five regression models, with TNL as the independent variable x and EPC as the dependent variable y, the goodness-of-fit R^2^ was used to assess the model fitting effect.

In the construction of energy consumption models, R^2^ is often chosen as the main criterion for model evaluation [19,23]. In this work, R^2^ was used to quantify the proportion of variance in electric power consumption (EPC) that was explained by the total nighttime light (TNL) as an independent variable, thereby serving as a direct indicator of the model’s explanatory capacity [27]. This approach aligns with our objective of identifying models that most effectively capture the relationship between TNL and EPC by maximising the explanatory capacity of TNL on EPC.

Although R^2^ prioritises the model’s explanatory power, it may artificially increase with the addition of predictors, even if they do not contribute significantly to the model’s explanatory capacity. To avoid spurious increases in R^2^, our model maintained simplicity (e.g., univariate power functions), which helped to ensure robust model performance. This is critical in spatiotemporal studies, where the complexity of spatiotemporal data (e.g., nonlinear relationships and heterogeneity) necessitates models with greater flexibility to capture these nuances.

R^2^ is a dimensionless coefficient with values ranging from 0 to 1. An R^2^ close to 1 indicates that the model predictions are closer to the actual results, suggesting a better fit.

To obtain the optimal fitting model applicable to the Chinese region, the electricity consumption estimation models in different space, time, and fitting dimensions were compared, respectively:

(1) Annual TNL–EPC spatial regression modelling, including the following: graded and ungraded.

The TNL of each province in a given year was taken as the independent variable, while the annual electricity power consumption (EPC) of the corresponding province was used as the dependent variable. During the fitting process, it was found that the samples from Heilongjiang, Tibet, and Xinjiang significantly deviated from the overall data trend. These regions were characterised by a high total amount of TNL but a low amount of EPC. This result suggests that these regions may have non-representative lighting sources that do not correspond to typical electricity consumption patterns. This deviation is probably because the high-intensity pixels in these areas have not been completely removed. This resulted in significant anomalies in the relationship between TNL and EPC in Xinjiang, Tibet, and Heilongjiang. The main reasons for the anomalies in these regions are as follows:

First, the anomalies in Xinjiang can be mainly attributed to extensive oil and gas extraction activities. Industrial operations such as drilling sites and flaring produce high-intensity lighting that significantly deviates from typical urban electricity consumption patterns. These industrial light sources produce abnormally high pixel values that distort the TNL–EPC relationship, thereby compromising model accuracy.

Second, the outliers in Tibet are related to the sparse population and low level of urbanisation. Lighting in the region is dominated by non-urban sources such as military infrastructure and aurora borealis. These sources of light are largely uncorrelated with social demand for electricity, resulting in a unique pattern that deviates from the general TNL–EPC relationship.

Finally, the anomalies in Heilongjiang are caused by frequent wildfires and agricultural burning activities, especially during the dry season. These activities result in transient high-brightness pixels that do not conform to typical urban lighting patterns. In addition, Heilongjiang has one of the highest rates of agricultural fires in China, especially in spring and fall. Crop straw burning significantly increases the nighttime light intensity, which further distorts the TNL–EPC relationship.

Provinces with significant outliers (e.g., Xinjiang, Tibet, and Heilongjiang) were excluded from the regression analysis. This step minimised bias due to non-representative light sources, such as industrial activities, wildfires, and non-urban infrastructure. With reference to previous studies, the generalisability of the model was ensured by masking these non-urban light sources, which, in turn, will improve the applicability of the model in different regions [20]. In the electricity consumption estimation model, R^2^ rose from around 0.5 to nearly 0.9 after excluding Xinjiang, and above 0.9 after excluding Tibet and Heilongjiang. Therefore, after excluding the 4 provinces with missing data and the 3 provinces with anomalous data, there were 27 provinces in total, and the spatial regression of the five models was fitted year by year. Then, the model with the largest R^2^ in each year was selected as the optimal model for that year.

On this basis, considering the differences in the levels of economic development of different regions, the light density of each province was calculated. The 27 provinces were divided into three levels according to the size of the light density, and the electricity consumption prediction models were constructed separately in the different levels to explore whether hierarchical fitting would improve the accuracy of the model. In 2012, for example, the grading results in three levels are presented in Table A1.

(2) Monthly TNL–EPC spatial regression modelling, including the following: graded and ungraded.

The TNL in December 2020, which had higher data quality and completeness, was selected as the independent variable. The corresponding monthly electricity power consumption (EPC) of each province was used as the dependent variable. During the fitting process, it was found that Shandong, Jilin, and Heilongjiang significantly deviated from the overall data trend. This deviation may be due to residual noise from non-urban light sources that was not completely removed, similar to the problems encountered in the annual regression model described above. As with Heilongjiang, the anomaly in Jilin was also mainly due to agricultural activities, particularly crop burning during the dry season, which introduces transient high-brightness pixels that distort the TNL–EPC relationship. Shandong, on the other hand, is an important industrial base in China (e.g., chemical, iron and steel, and manufacturing). The industrial facilities in Shandong may continue to operate at night, generating high-intensity, steady lighting signals that deviate from typical residential electricity consumption patterns. Such lighting inflates the total nighttime light (TNL) beyond what actual electric power consumption (EPC) can match due to differences in the way industrial electricity consumption is counted (e.g., captive power plants, which are not fully included in grid data). In addition, the nighttime lights of logistics operations in port cities, such as Qingdao and Yantai, may be misclassified as residential electricity consumption, leading to a deviation in the TNL–EPC relationship by inflating the TNL beyond what EPC can match, as documented in previous studies [19]. Therefore, after removing the 4 provinces with missing data and 3 provinces with data anomalies, regression fitting was performed on a total of 27 provinces, and the model with the highest R^2^ was selected as the model with the best fitting effect in December 2020. Similar to the construction of the spatial dimensional hierarchical model for annual data, the provinces were graded, and the electricity consumption prediction models were constructed separately. In addition, the fitting effects before and after grading were compared.

(3) Annual TNL–EPC temporal regression modelling, including on the national, provincial, and prefectural scale.

The TNL data for the nation, provinces, and major prefectures were collected annually from 2012 to 2023 as the independent variable. The corresponding electricity power consumption (EPC) of each administrative unit (national, provincial, and major prefectural levels) was used as the dependent variable. A total of 12 annual sample points for each administrative unit were used for regression fitting. The model with the highest R^2^ value was selected as the best-fitting model in the temporal dimension. In the construction of prefectural scale electricity consumption models, it was difficult to model all cities in China in practice. For this reason, electricity consumption models for the capital cities of each province in China were developed in this work. In this modelling, excluding the areas with missing data, 30 provinces and 18 cities were finally modelled.

(4) Monthly TNL–EPC temporal regression modelling, including one the national, provincial, and prefectural scale.

The TNL data for the nation, provinces, and major prefectures were collected monthly in 2020 and used as the independent variable. The corresponding monthly EPC of each administrative unit was used as the dependent variable. After excluding months with missing data, a total of seven monthly sample points per administrative unit were used for regression fitting. Due to some difficulties in the actual collection of electricity consumption data for prefecture-level cities, a total of 30 provinces and 9 cities were finally modelled.

### 3.2. Spatialisation of China’s Electricity Consumption Data

The optimal electricity consumption estimation model obtained from the comparison was used to calculate China’s electricity consumption over the last 12 years. By providing the independent variable TNL, the dependent variable EPC was derived. Then, the spatialisation of the electricity consumption data was completed by performing raster calculations, and the spatialised electricity consumption data product of China over the past 12 years was obtained.

## 4. Results

### 4.1. Electricity Consumption Nighttime Light Estimation Modelling Results

To obtain the most suitable electricity consumption estimation model for China, annual and monthly TNL data were, respectively, used to fit with the electricity consumption data in the spatial and temporal dimensions.

(1) Spatial regression results.

In the annual data spatial dimension electricity consumption modelling, in the regression results for each year from 2012 to 2023, the R^2^ gradually decreased in the order of power function model, quadratic polynomial model, linear model, exponential model, and logarithmic model. This implies that the fitting effect decreased in this order. The power function model exhibited the best fit, reaching over 0.9, and the logarithmic model had the worst fit. Table 2 provides the regression results for each electricity consumption model for 2023.

Then, the provinces with different light densities were graded. When dividing the densities into three grades, the regression effect became worse as the city grade decreased, that is, as the light density decreased. The regression effect was the best in the first-level regions, with R^2^ close to 1; this was improved compared with that when the light densities were not divided into grades. The regression effect of the second-level and third-level regions became worse compared with that when the light densities were not divided into grades. In addition, the fitting effects under different classification numbers, such as two levels, four levels, and five levels, were also investigated. The results indicated that within a certain limit, as the number of levels increased, the fitting effect of high-level areas gradually improved. Table 3 shows the regression results of the electricity consumption models for the different classes, with three classes as an example.

For the monthly data, the fitting effect decreased in the order of quadratic polynomial, linear, power function, logarithmic, and exponential models. The quadratic polynomial model worked best with a goodness of fit of 0.9381, and the exponential model had the worst fit. Table 4 shows the regression results for December 2020 for each electricity consumption model.

Similar to the annual data, when the monthly data of provinces with different light densities were classified into three levels, the fitting effect of high-level regions was improved; the R^2^ of first-level regions increased to 0.961. Although the fitting effect of level 2 and level 3 regions decreased, the R^2^ values of level 2 and level 3 regions were also significantly improved after removing the individual anomalies; the R^2^ value of level 3 areas reached 0.9477. The fitting effects under different numbers of classifications, such as two levels, four levels, and five levels, were investigated. The results were similar to the spatial dimensional hierarchical fitting of the annual data, showing a pattern of increasing classification levels and improved fitting effects in high-level regions.

(2) Temporal regression results.

In the annual data temporal dimension electricity consumption model construction, under the national scale, the best fit was the quadratic polynomial model with an R^2^ of 0.9706; the logarithmic model was the worst with an R^2^ of 0.9519. Table 5 demonstrates the regression results of the national electricity consumption model in the temporal dimension. At the provincial scale, except for Tibet and Qinghai, the provinces fit better, with Heilongjiang having the lowest goodness-of-fit at 0.7485 and Chongqing having the highest goodness-of-fit at 0.9898. Table A2 shows the optimal regression results for each provincial fit in descending order. At the city scale, the fitting results of major cities were very good, with Harbin having the lowest goodness-of-fit at 0.8224 and Changsha having the highest goodness-of-fit at 0.9921. Table A3 shows the optimal regression results of major cities in descending order.

Overall, the temporal regression models for annual data achieved a good fit at all spatial scales, except for Tibet and Qinghai.

In the temporal regression on monthly data, the fitting effect was not good under the national scale; the maximum goodness-of-fit was 0.4667 for the quadratic polynomial model. Under the provincial scale, the regression results for each province were mixed; the provinces with good fitting effects included Jilin, Heilongjiang, etc.; the provinces with average fitting effects included Hebei Province, Shandong Province, etc.; the provinces with poor fitting effects included Zhejiang, Anhui, etc. Among them, the fitting effects of the linear, exponential, logarithmic, and power function models in Beijing, Tianjin, Jiangsu, Shanxi, Henan, Hainan, Chongqing, Shaanxi, and Gansu were very poor. Nonetheless, the impact of the quadratic polynomial model fitting was improved; although the fitting effects of Jiangsu, Guizhou, Hainan, Yunnan, Tibet, and Qinghai were better, the linear fitting curves showed that with increases in the TNL, the electricity consumption decreased. This result is consistent with Zhang et al.’s finding of a negative correlation between TNL and EPC during this period [28]. This confirms the plausibility of our results. Table A4 shows the descending order of the optimal regression results for each province.

The prefectures with better fitting results included Nanjing, Hohhot, Harbin, and Taiyuan. The regression results for the linear, exponential, logarithmic, and power function models in Shenyang, Nanjing, and Haikou were poor, but the fitting effect of the quadratic polynomial model was improved; the linear regression models for Jinan, Nanjing, and Zhengzhou appeared to exhibit a decrease in electricity consumption with increases in the TNL. Table A5 shows the descending order of the optimal regression results for different cities.

Contrary to the widely reported positive TNL–EPC correlation, negative correlations in both the provincial-level linear models (e.g., Jiangsu, Hainan Province) and municipal-level linear models (e.g., Jinan, Zhengzhou) were found for the monthly temporal regression model. This may be related to the local climate (e.g., seasonal peaks in electricity consumption, winter haze vs. cloudy summer in Zhengzhou), special economic activities (e.g., dependence on off-grid power supply in Tibet and Qinghai), and policies and energy mixing (e.g., Hainan’s energy efficiency policy reduces lighting but increases EV charging).

Moreover, the monthly data contained only seven months, which resulted in random fluctuations due to the small sample size. Regions with low signal-to-noise ratios (e.g., Qinghai, Tibet) were particularly prone to spurious negative correlations. Linearity failed to capture the dynamics of the TNL–EPC relationship, leading to fitting bias. These findings challenge the assumption of a general positive correlation between TNL and EPC and highlight the need for region-specific calibration in nighttime lighting models.

As a result, the monthly data temporal regression model was only able to achieve better results in individual provinces and prefectures, and overall, it was less effective at the national, provincial, and prefectural levels.

### 4.2. Results of Spatialisation of Electricity Consumption

As an example, the national-scale electricity consumption estimation model for the year 2023 from the temporal regression results in Section 4.1 was used to estimate and spatialise the electricity consumption using the following regression equation:

y = 3E−11x^2^ + 0.0015x + 25,625 (1)

**Figure 2:**
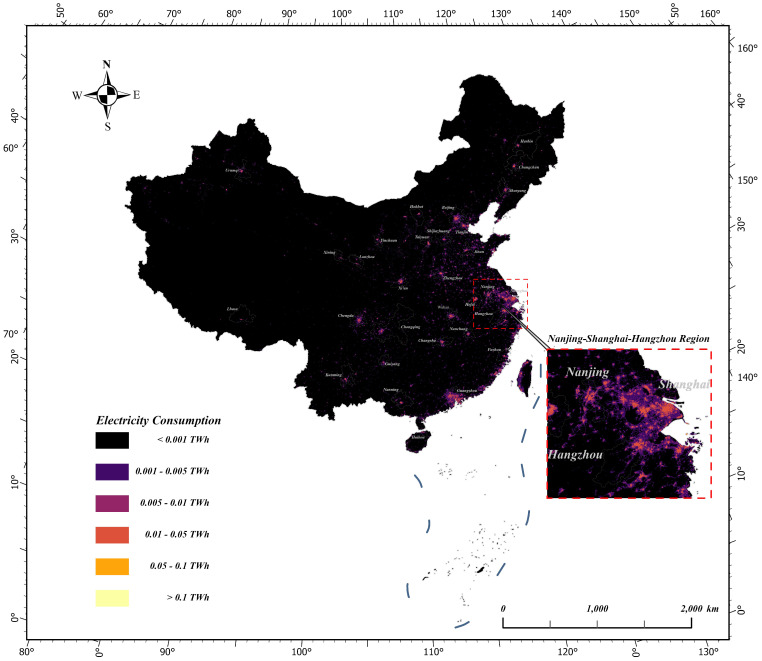
Spatialisation of the temporal dimension electricity consumption model in 2023.

The estimated electricity consumption (Unit: TWh) was divided into six categories: less than 0.001, 0.001 to 0.005, 0.005 to 0.01, 0.01 to 0.05, 0.05 to 0.1, and greater than 0.1. The spatialised results are shown in Figure 2.

## 5. Discussion

### 5.1. Comparison of the Spatial Regression Results

#### 5.1.1. Comparison of the Provincial Spatial Regression Results for Annual Data Without and with Grades

When comparing the optimal regression results before and after grading, it was observed that the R^2^ value can exceed 0.9 without grading. However, grading has differential impacts on the fitting effects of high- and low-level regions. More specifically, the fitting effect of high-level regions was improved after grading, while that of low-level regions deteriorated. This result suggests that a higher light density is associated with better fitting effects and a higher estimation accuracy of electricity consumption. Grading can significantly enhance the fitting effect of high-level regions, likely due to the more consistent relationship between the light density and electricity consumption in these areas.

The threshold method used in this work for extreme value removal is based on reference data from high light density areas, such as Beijing and Shanghai. Consequently, more extreme values remain in regions with lower light density compared to high light density regions. This leads to a greater impact on the fitting effect in low light density regions, resulting in a poorer overall fit. This finding highlights the challenges in applying a uniform threshold method across regions with varying light densities and suggests the need for the development of region-specific approaches to improve model accuracy.

Zhang et al. combined nighttime lighting with GDP and population data for county-level EPC estimation (R^2^ = 0.91) [22]. Their results outperformed our hierarchical function model in underdeveloped areas (R^2^ = 0.844). This suggests that while our model, relying solely on nighttime lighting, can achieve comparable accuracy in rural areas, integrating multiple variables can significantly enhance model performance, particularly in underdeveloped areas.

In summary, grading improves the fitting effect of high-level regions but has a negative impact on low-level regions, highlighting the importance of considering regional differences in model development. The threshold method used in this work is effective for high light density areas but needs refinement for low light density regions. Future research should explore region-specific threshold methods and incorporate additional variables to further enhance model accuracy across different regions.

Therefore, when using annual data for the spatial regression of provinces, considering the overall fitting effect, the power function model should be used to model provinces non-hierarchically. Table 6 shows the regression results before and after grading in 2012 and 2023.

#### 5.1.2. Comparison of the Provincial Spatial Regression Results for Monthly Data Without and with Grades

By comparing the optimal regression results before and after grading, we found that a higher light density in the regions led to a great improvement of the fitting effect after grading, the same as observed for the annual data. However, the monthly data often had outliers in regions with low light densities, which led to less stable regression effects in low-level areas.

Therefore, when using monthly data for the spatial regression of provinces, after reasonably eliminating outliers, the impact of using graded fitting in some months will be better than when there is no grading. Nonetheless, the impact of direct fitting is more stable, and a quadratic polynomial model R^2^ around 0.9 is generally observed. Table 7 shows the regression results before and after grading in December 2020 as an example.

#### 5.1.3. Comparison of the Provincial Spatial Regression Results for Annual and Monthly Data

Combining the regression results in the spatial dimension, when annual data are used, good electricity consumption prediction results can be obtained by directly building power function models for each province. On the contrary, when monthly data are used, it is necessary to decide whether or not to construct the model in a hierarchical manner according to the sample data. The utilisation of a quadratic polynomial model in the case of the no hierarchical manner can yield more stable results. Most of the R^2^ values in the spatial dimension for annual data were above 0.9, while most of the monthly data were around 0.9, which is in line with the reality that the quality of annual data is better than that of monthly data.

Therefore, to obtain and spatialise China’s electricity consumption in the period of 2012–2023, the provincial power function spatial regression model should be constructed directly using annual data.

#### 5.1.4. Spatial Regression Model for China’s Electricity Consumption, 2012–2023

The construction of power function models using annual data and the corresponding function for each year are shown in Table A6. Table A7 shows the results of electricity consumption estimated using the model in Table A6. In this table, the largest relative error was −5.97%, the smallest was −1.31%, and the average relative error was −1.71%, which demonstrate the feasibility of estimating China’s electricity consumption in the spatial dimension using the total annual nighttime light.

Compared to previously reported studies where provincial electricity usage has been modelled, our power function model had an average R^2^ value of 0.93, which is better than the linear and logarithmic models commonly used in earlier studies. This represents a significant improvement over He et al.’s [18] provincial electricity modelling (2000–2008) using a linear model (R^2^ = 0.85) and Shi et al.’s [19] global logarithmic model using calibrated DMSP-OLS data (R^2^ = 0.89).

In addition to benefiting from the comparative advantages of the NPP-VIIRS data, which enabled us to accurately detect light variations in the urban core and peri-urban areas, the removal of biases from anomalous provinces (e.g., Xinjiang, Tibet, and Heilongjiang) was also an important reason for obtaining better modelling results. These biases, driven by unique local factors, such as industrial activities and agricultural burning, resulted in non-representative light patterns.

Moreover, the quadratic term in the power function was able to adaptively capture sub-linear trends in mature cities (e.g., Shanghai) and super-linear growth in emerging centres (e.g., Chongqing). This flexibility allowed the model to better capture the observed spatial heterogeneity in China’s electricity consumption patterns, where densely populated areas (e.g., coastal cities) showed diminishing marginal growth in per-unit EPC and TNL, while rapidly industrialising areas showed accelerating growth.

### 5.2. Comparison of the Temporal Regression Results

#### 5.2.1. Comparison of the Temporal Regression Results at Different Spatial Scales for Annual Data

Combining the results in Table 5, Table A2, and Table A3, the temporal regression models of annual data at different spatial scales showed high effectiveness, except in Tibet and Qinghai, where excessive noise resulted in almost no correlation between TNL and EPC. The results indicated that the fitting effect varied across regions, suggesting that regional differences, rather than spatial scale size, are key factors affecting model performance. For example, our model performed poorly in regions with weak TNL–EPC correlations, such as Heilongjiang. These gaps highlight the need for multi-source data fusion in future work, especially in regions where the TNL–EPC correlation is weak, to enhance the model’s accuracy and robustness.

In Table A6 for the provincial and Table A7 for the municipal temporal regression results, eight provinces and three prefectures had a goodness-of-fit greater than 0.9706. This suggests that no more than one-third of the 30 provincial and one-sixth of the 18 prefectural temporal regressions, respectively, had an improved fit compared to the national temporal regression results. Considering the overall fitting effect, the quadratic polynomial model appears to be the most suitable method for estimating nationwide electricity consumption in China when using annual data for temporal regression.

#### 5.2.2. Comparison of the Temporal Regression Results at Different Spatial Scales for Monthly Data

When monthly data were used to estimate China’s nationwide electricity consumption, the temporal regression model did not work well, but it worked better in some provinces and prefectural scales. By analysing the regression results for each province in Table A4, it was found that a small number of regions located in the north of China had very good fitting effects. Moreover, most of the regions had fair fitting effects; these regions were mainly distributed in the central part of China. There were many other regions with very poor fitting effects; these were mainly distributed in the south of China. In the latter regions, the data were almost uncorrelated, which may be due to the fact that there are a lot of clouds in the southern region. This leads to a poorer quality of nighttime light data compared with the other regions, which affects the fitting effect. Overall, the regions showed a decreasing fitting effect from north to south according to geographic location. Interestingly, although the sample size was small, the regression results for each city in Table A5 were geographically consistent with those in Table A4. It can be also argued that the fitting effect was better in the northern region than in the southern region, which further confirms our conjecture that the quality of the monthly data was deteriorating in the southern part of China.

Therefore, when using monthly data for temporal regression within China, the size of the spatial scale has no effect on the modelling of electricity consumption. In striking contrast, the quality of the nighttime light data caused by the geographic location is one of the factors affecting the fitting effect, and the further north the geographic location, the higher the quality of nighttime light data. A higher quality of nighttime light data leads to a better fitting effect.

#### 5.2.3. Comparison of the Temporal Regression Results for Annual and Monthly Data

Synthesising the regression results in the temporal dimension, when annual data were used, a quadratic polynomial model at the national scale yielded the optimal fit; when monthly data were used, a quadratic polynomial model at the spatial scales of provinces and prefectures provided a better fit for northern China. The fitting effect of the annual data in the temporal dimension was significantly better than that of the monthly data. This effect, on the one hand, can be ascribed to the better data quality of annual data than that of monthly data. On the other hand, the sample size of monthly data was smaller, which could result in the obvious regional differences in the monthly data and the very poor fitting effect in large spatial scales.

Therefore, when performing model building in the temporal dimension, to obtain and spatialise China’s electricity consumption from 2012 to 2023, a national quadratic polynomial temporal regression model should be constructed using annual data.

#### 5.2.4. Temporal Regression Model for China’s Electricity Consumption, 2012–2023

A quadratic polynomial model was constructed using annual data. Table A8 presents the results of using the quadratic polynomial model to predict the electricity consumption. As can be observed, the largest relative error was 9.56%, the smallest was 0.76%, and the average relative error was 2.86%. These results indicate that in the temporal dimension, the utilisation of the total annual nighttime light to estimate China’s electricity consumption is a feasible approach.

The quadratic polynomial model (R^2^ = 0.9706) revealed an acceleration in electricity consumption in China after 2018. This is distinct from the global trend and contrasts with the linear relationship between TNL and EPC reported by Falchetta et al. for low- and middle-income countries (R^2^ ≈ 0.82) [23]. Their methodology may underestimate the nonlinear surge in electricity consumption driven by China’s rapid renewable energy expansion after 2018. The observed nonlinear acceleration coincides with China’s post-2018 push for renewable energy, which has increased grid complexity and the nighttime lighting of wind and solar farms. This highlights the importance of high temporal resolution data (e.g., annual vs. decadal) and region-specific policy contexts in energy modelling.

Quadratic polynomial models are particularly well suited to capturing complex growth patterns, especially accelerated growth phases, compared to linear and exponential models. This capability is crucial for accurately representing the dynamics of electricity consumption. The quadratic term specialises in capturing potential accelerating or decelerating consumption patterns, which can introduce variability and non-stationarity into the TNL–EPC relationship.

As a result, quadratic polynomial models exhibited a significant advantage in capturing the accelerated growth of electricity consumption in China after 2018. For example, Townsend and Bruce [20] used an exponential model (R^2^ = 0.75) but failed to accurately capture the deceleration phase of electricity consumption in industrial areas. This highlights the limitations of exponential models in handling complex consumption patterns, particularly in regions with significant industrial activity.

The pattern of electricity consumption varies considerably across countries and regions due to factors such as ecological and economic structures, energy policies, and technological advances. Therefore, model selection should be guided by the specific characteristics of the data and the underlying economic and policy contexts to ensure accurate and reliable results.

Our quadratic term explicitly modelled the compounding effect of China’s energy transition. More specifically, the construction of wind and solar farms after 2018 increased nighttime lighting for infrastructure maintenance, which decoupled TNL growth from traditional electricity consumption drivers. As renewable energy projects advanced, nighttime lighting demand increased, but these additions are not fully reflected in the growth of traditional electricity consumption, leading to a nonlinear relationship between TNL and EPC.

In addition, the quadratic curve reflected the delayed effect of China’s 2015 “coal to clean energy” policy, which initially dampened electricity consumption but gradually accelerated it as the grid stabilised. Quadratic terms can effectively capture this nonlinear change, which linear or exponential models cannot.

### 5.3. Comparison of the Spatial and Temporal Regression Results

In Section 5.1 and Section 5.2, the optimal estimation models of China’s electricity consumption in the spatial and temporal dimensions were obtained, respectively. In terms of goodness of fit, the spatial dimension was fitted using a power function model, with an average R^2^ of 0.9385, while the temporal dimension was fitted using a quadratic polynomial model, with an average R^2^ of 0.9706; this gave a better fit in the temporal dimension. In terms of relative error, the maximum error in the spatial dimension was −5.97%, the minimum was −1.31%, and the average relative error was −1.71%, while the maximum error in the temporal dimension was 9.56%, the minimum was 0.76%, and the average relative error was 2.86%; this indicates a better fit for the spatial dimension.

**Figure 3:**
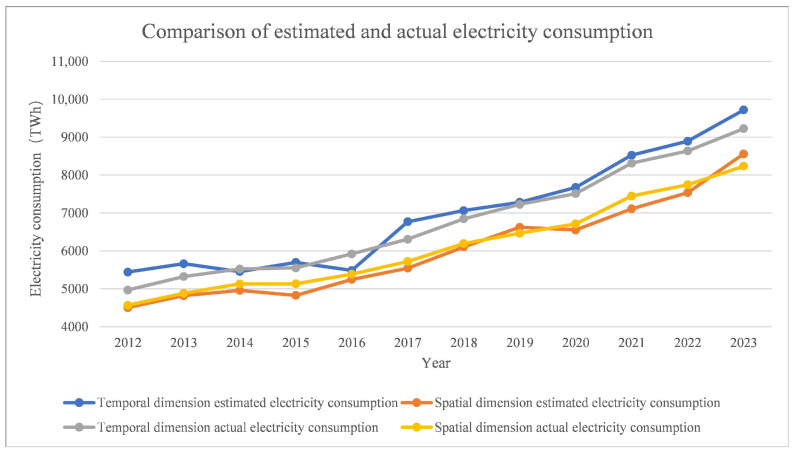
Comparison of estimated and actual electricity consumption, 2012–2023.

The predicted and actual electricity consumption of the two dimensions were plotted in a line graph, as depicted in Figure 3. By comparing the spatial dimension predicted electricity consumption with the actual electricity consumption, an overestimation in 2023 and an underestimation in 2015 and 2021 can be observed, with an average relative error of −1.71%. The overall performance of the underestimation and the trend was the same as the actual electricity consumption; for the temporal dimension, there were overestimates in 2012, 2013, 2017, 2021, 2022, and 2023, and an underestimate in 2016. The average relative error was 2.86%, the overall performance was overestimated, and the trend was consistent with the actual electricity consumption.

To further explore the advantages and disadvantages of the electricity consumption models constructed under the two dimensions, all regression results for the spatial and temporal dimensions were compared. Table 8 demonstrates all the model construction results for the spatial and temporal dimensions.

First, the table was compared horizontally. In the annual TNL–EPC model, the fitting effect of the temporal dimension was better, and in the monthly TNL–EPC model, the spatial dimension was fitted better. This was mainly manifested in the fact that the fitting effect of the spatial dimension was more stable; vertically, whether it is the temporal dimension or the spatial dimension, from annual data to monthly data, the decline in the quality of the data resulted in a weakening of the two dimensions’ fitting effects. However, the fit of the spatial dimension was more stable than that of the temporal dimension, and the temporal dimension was fitted better than the spatial dimension under the condition of good data quality.

In summary, the fitting of the spatial dimension was more tolerant to the decrease in the fitting effect caused by the decrease in the quality of the nighttime light data. Hence, good results when modelling lower-quality monthly data can be also obtained, while the fitting of the temporal dimension has a better fitting effect than the spatial dimension when the data quality is good. It is, thus, more suitable for use in the case of high data quality. In addition, the fitting of the spatial dimension uses a smaller amount of data than the temporal dimension. Nonetheless, high refinement of electricity consumption data is required, and it is prone to produce areas with missing data, for which electricity consumption estimation is not possible. In contrast, the fitting of the temporal dimension generally targets all regions in a range, and the electricity consumption data are more easily accessible. Therefore, when there are some regions in a range that have missing data, but the total data of the range are available, it can make up for the estimated electricity consumption in the regions with missing data.

In summary, the spatial and temporal dimensions of China’s electricity consumption estimation models have their own advantages in different situations, and the most suitable electricity consumption model for spatialisation should be selected based on the actual needs.

### 5.4. Analysis of Time–Space Variation in China’s Electricity Consumption, 2012–2023

#### 5.4.1. Spatialisation of Electricity Consumption in China

**Figure 4:**
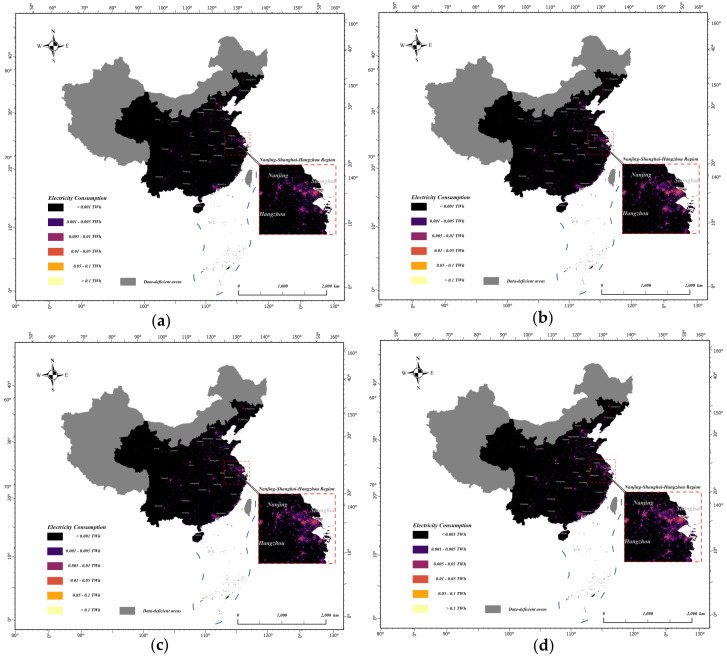
Spatial distributions of electricity consumption in China in the spatial dimensions of (**a**) 2012; (**b**) 2016; (**c**) 2020; and (**d**) 2023.

**Figure 5:**
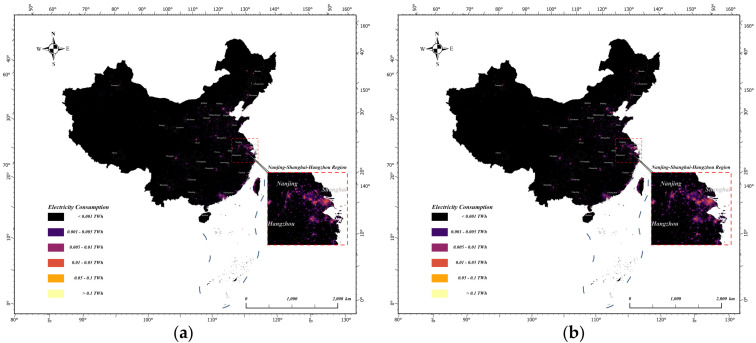

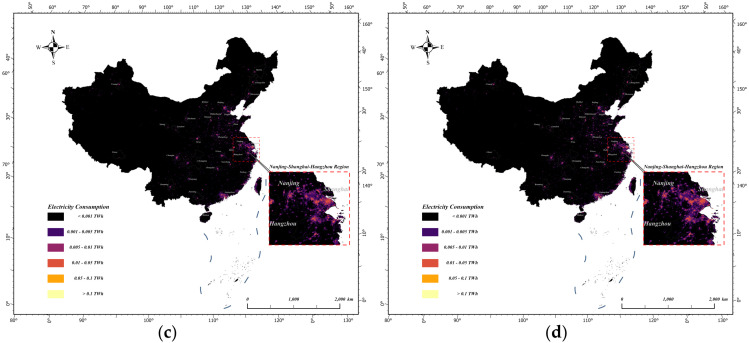
Spatial distributions of electricity consumption in China in the temporal dimensions of (**a**) 2012; (**b**) 2016; (**c**) 2020; and (**d**) 2023.

The optimal electricity consumption estimation model with spatial and temporal dimensions in Section 5.1 and Section 5.2 was used to spatialise China’s electricity consumption for the last 12 years. Then, the spatialised Chinese electricity consumption data products with a 500-metre resolution were obtained. Taking 2012, 2016, 2020, and 2023 as examples, Figure 4 and Figure 5 show the results of the spatialisation of China’s electricity consumption in the spatial and temporal dimensions, respectively.

From the results of the spatialisation of electricity consumption in both dimensions, the spatialisation of the spatial dimension better reflected regional differences, while the spatialisation of the temporal dimension better reflected temporal changes in electricity consumption. Combined with the discussion in Section 5.3, there are advantages and disadvantages of modelling using the temporal and spatial dimensions, and thus, each has its own appropriate electricity consumption model for different application requirements and data situations.

For research purposes, the optimal models in both the spatial and temporal dimensions can be used to produce spatialised electricity consumption data for China for the last 12 years.

#### 5.4.2. Time–Space Variation in China’s Electricity Consumption

Using spatialised electricity consumption data to analyse the time–space variation in China’s electricity consumption, it can be seen that overall, China’s electricity consumption has continued to increase over the past 12 years. From 2012 to 2023, basically centred on the capital city of each province, the number of areas with high electricity consumption has been increasing and the scope has been expanding. This result is shown in the graph with the bright areas becoming heavier in colour and the scope gradually expanding. The increase in electricity consumption is shown in the graph as a gradual development from point to surface and a gradual connection between regions from point to line. The continuity between the regions is enhanced, and regions with high electricity consumption are connected with each other to form a cluster of high-electricity-consumption cities.

From a geographical point of view, electricity consumption was higher in the eastern region than in the central region and the western region, and higher in the coastal region than in the inland region. A large difference existed between the east and west, and this exhibited a certain correlation with the distribution of China’s topography. High electricity consumption regions were concentrated in the Beijing–Tianjin–Hebei region (centred on Beijing and Tianjin, with Tangshan, Langfang, etc. as sub-centres), Yangtze River Delta (centred on Shanghai, with Nanjing, Hangzhou, etc. as sub-centres), and Pearl River Delta (centred on Guangzhou and Shenzhen).

To summarise, the regions with higher power consumption are mostly located in the coastal areas and inland industrial areas, mainly including the Beijing–Tianjin–Hebei region, Yangtze River Delta region, Pearl River Delta region, provincial capital cities, and sub-provincial cities in the eastern coastal region, such as Dalian, Qingdao, Ningbo, Quanzhou, and so on.

#### 5.4.3. Changes in Electricity Consumption in Major Chinese Prefectures

To gain a deeper understanding of the time–space variation in China’s electricity consumption, the results of the spatialisation of the optimal electricity consumption model in the temporal dimension were used to obtain data on the electricity consumption of China’s major prefectures. Table 9 and Table 10 show the top 10 and bottom 10 rankings of the incremental and growth rates of electricity consumption for China’s major prefectures over the past 12 years.

By comprehensively analysing the electricity consumption data of each prefecture over the past 12 years, it can be seen that the regions with the leading increment in electricity consumption were mainly big cities with an active economy, such as Chongqing, Wuhan, Beijing, etc. These cities are lighter in colour in the distribution map of electricity consumption in all years. In terms of the growth rate of electricity consumption, the growth rate of these prefectures was not at the forefront, with most around 5%, and the electricity consumption presented steady growth at a relatively low rate. This result indicates that the development of these cities is entering a saturation period. However, due to the large base of electricity consumption, they are the mainstay of electricity consumption in the country.

Cities with less incremental electricity consumption, or even with an incremental increase close to 0, were mainly located in less populated areas such as Hainan, Xinjiang, and Tibet. These are generally more remote and far from economic centres, with a small base of electricity consumption. From the growth rate of electricity consumption, the growth rates of some prefectures in Hainan and Xinjiang, such as Baoting, Qiongzhong, Kunyu, and Huyanghe, were higher, which indicates that these areas are developing gradually. The increments and growth rates of Yan’an and Wuzhishan were close to zero, indicating a certain level of stagnation; these cities need to seek a new direction for development.

The areas with negative increments were Daqing, Fushun, Benxi, Panzhihua, and Liaoyang, among which, Daqing, Fushun, Benxi, and Liaoyang are typical old industrial cities in northeast China. In these regions, secondary industry accounts for the majority of economic output. Panzhihua is the same as the above-mentioned areas; it relies on the industrial production of mineral mining and metal smelting to develop its economy. In addition, the petrochemical industry, the coal industry, and the electric power industry take up a large amount of electric power consumption. These cities’ negative growth in electricity consumption is the result of a positive response to the transformation of the country’s heavy industrial cities. In terms of the growth rate of electricity consumption, the result and the increment were uniform, reflecting the positive transformation of China’s heavy industrial cities.

Over the past 12 years, the regions with the top growth rates of electricity consumption were mainly located in Hainan, some cities in Xinjiang, and small cities in the southern region. This is partly due to the fact that the development speed of large cities has reached saturation. As a result, some small cities in the southern region have begun to develop. This finding is also partly due to the results of the nation’s active regulation. For example, the results for Xinjiang fully demonstrate that China is currently actively adjusting the current status of imbalance and insufficient development, and has achieved certain results. In terms of increment, the minimum increment of these cities was 0.2 TWh, the maximum was around 20 TWh, and the increments of most of the cities were between 5 and 15 TWh, which is the reserve for future electricity consumption.

The areas with smaller growth rates of electricity consumption mainly included small cities in the central region such as Xinzhou, Handan, Jiuquan, Yan’an, etc. The development of these cities may have reached a bottleneck; new development strategies need to be actively deployed in the context of local realities. In addition, the growth rates of electricity consumption in large economically developed cities such as Shanghai, Suzhou, Tianjin, etc. exhibited slowing and were gradually saturated. In terms of increment, the incremental increases were small in small cities and large in economically developed cities.

From 2012 to 2023, there were 29 major cities in China with an increase in electricity consumption of more than 30 TWh, 31 cities with 20 to 30 TWh, 96 cities with 10 to 20 TWh, and 203 cities with 0 to 10 TWh, among which, there were 29 cities with an increment of 0 to 2 TWh and 5 cities with a negative increase. The overall performance of the cities indicates that a few areas use most of the electricity and the problem of imbalance in electricity consumption is prominent. The growth rate of electricity consumption was above 15% in a total of 8 cities, 10–15% in a total of 74 cities, 5–10% in a total of 194 cities, and 0–5% in a total of 81 cities, among which, the rate was less than 2% in a total of 12 cities and the growth rate was negative in a total of 5 cities. In the future, areas with growth rates ahead of the others, especially in the southern region of small cities, will become high power consumption areas.

The expansion of high-consumption industrial clusters (e.g., Yangtze River Delta, Pearl River Delta) echoes the “core-periphery” theory of regional development [29], but with two distinct features:

(1) The growth of third-tier cities. Unlike the previously reported studies that emphasised megacities [19], our findings highlight the rise of third-tier cities in the south (e.g., Yibin, Wenzhou) as new consumption centres. While Friedman predicted centralised growth in core regions, our findings suggest that China’s “county-level urbanisation” policy, which disperses infrastructure investment to ease the burden on megacities, is driving decentralised growth.

(2) The decline of industrial cities. The negative consumption growth in EPCs in northeastern industrial bases (e.g., Daqing, Fushun) contrasts with stable patterns in post-industrial Europe [23], reflecting China’s aggressive decarbonisation policies since 2015. Unlike Europe’s retraining programs, China’s mass migration of laid-off workers to southern cities has accelerated regional consumption divergence.

These findings not only extend the core–periphery theory by highlighting the need to adapt the classical framework to rapid policy shifts in developing economies but also provide empirical evidence of policy-driven consumption fragmentation.

### 5.5. Factors Affecting the Effectiveness of Model Fitting and Limitations of This Paper

#### 5.5.1. Factors Affecting the Effectiveness of Model Fitting

(1) Data quality. In the fitting of the spatial and temporal dimensions, the annual data fit much better than the monthly data; in the temporal dimension, the monthly data fit better in the north than in the south. Together, these findings indicate that the quality of the nighttime light data affects the effectiveness of model fitting.

(2) Denoising methods. In the fitting of spatial dimensions, after grading the data, the fitting effect was improved in high light density areas, while it decreased in most regions of low light density. Notably, removing data from Xinjiang significantly enhanced the fitting effectiveness. This may be due to the denoising method focusing on high consumption regions like Beijing and Shanghai. This suggests that background noise may exist more in low consumption areas. Therefore, changing the denoising method for these regions could enhance model fitting.

(3) Regional differences. The development of different regions is distinct. The fitting to temporal dimensions varied between the annual and monthly data across provinces and prefectures. Therefore, when constructing the model for different regions, it is crucial to consider various influencing factors to enhance model fitting.

(4) Fitting method. In general, fitting in the spatial dimension was more likely to yield better model-building results than in the temporal dimension. This dimension is more tolerant of the effects of data quality and regional differences on fitting effectiveness.

#### 5.5.2. Limitations

(1) The method of denoising noise from NPP-VIIRS nighttime light data used in this study was not effective for low electricity consumption areas. As a result, there was still a certain amount of noise in the processed nighttime light data, which may have affected the accuracy of the electricity consumption estimation.

(2) In constructing the model, the electricity consumption was for the whole of society. Further research on the potential for enhancing the accuracy of electricity consumption estimates through the modelling of industrial, residential, or sub-industrial electricity consumption is needed.

(3) Due to the difficulty of collecting electricity consumption data, the small number of samples involved in modelling in the study likely affected the accuracy and reliability of the electricity consumption prediction to a certain extent.

(4) Although the nighttime light data showed a good correlation with the electricity consumption data overall, there are limitations in using a single data source for regions with significant regional differences, low electricity consumption areas, or areas affected by environmental disturbances. It is important to incorporate a variety of influencing factors, such as the impact of population and industry, to enhance the accuracy of electricity consumption estimations.

(5) This work investigated only the relationship between the nighttime light data and electricity consumption data. The introduction of other complementary data sources, such as utility infrastructure data and renewable energy production statistics, could have improved the model’s scalability and accuracy.

(6) While the exclusion of some anomalous provinces (e.g., Xinjiang, Tibet), which exhibited non-representative lighting patterns due to unique local factors, improved the model fit in the spatial dimension, this approach has significant limitations. More specifically, it underrepresents remote regions and energy-intensive areas, creating geographic coverage disparities and economic activity discrepancies. These issues may compromise the model’s generalisability and accuracy in these underrepresented areas, limiting the model’s ability to accurately reflect the true relationships in the spatial dimension.

## 6. Conclusions and Recommendations

In this work, an electricity consumption estimation model using NPP-VIIRS nighttime light data and China’s electricity consumption data across different administrative units was constructed. Our objectives were to identify the optimal fitting models for China in both the spatial and temporal dimensions, generate a spatialised map of China’s electricity consumption over the past 12 years, and analyse the spatiotemporal variations in electricity consumption. The main conclusions and recommendations are as follows:

(1) In the spatial dimension, the power function model is considered the optimal fitting model, with an average R^2^ of 0.9385. In the temporal dimension, the quadratic polynomial model is considered the optimal model, with an R^2^ of 0.9706. These results demonstrate the effectiveness and reliability of using total nighttime lights to estimate electricity consumption.

(2) The spatial regression model effectively highlights the regional differences in electricity consumption, revealing distinct patterns across various areas. Meanwhile, the temporal regression model captures the dynamics of electricity consumption changes over time, providing valuable insights into trends and fluctuations.

(3) The fitting effect of the electricity consumption estimation model is primarily related to data quality, denoising methods, regional differences, and fitting methods (e.g., linear, quadratic or exponential models), rather than the size of the spatial scale. High-quality data and appropriate methods can significantly improve the accuracy of the estimation and ensure that the model reliably reflects the real consumption patterns in different regions.

In large study areas, fixed thresholds fail to distinguish between industrial lighting (e.g., oil fields in Xinjiang), natural disturbances (e.g., wildfires in Heilongjiang) and valid residential signals. Developing dynamic adaptive noise filtering algorithms, such as machine learning-based dynamic thresholds and spatiotemporal interpolation of outliers, can address this issue. Integrating multisource nighttime lighting data, such as high-resolution data (e.g., Luojia-1, 130 metres) for enhanced urban detail and thermal infrared data (e.g., MODIS LST) to distinguish industrial heat sources from residential lighting can improve data quality by reducing misclassification of high-temperature facilities (e.g., steel plants).

For low-consumption regions, integrating multisource data, such as social media location data (nighttime activity levels) and traffic flow data, can significantly enhance model accuracy, especially in areas like Tibet and Qinghai. Developing low-light enhancement algorithms, such as deep learning-based super-resolution reconstruction, can improve spatial detail in low-brightness areas. Combining microwave remote sensing (e.g., Sentinel-1 SAR) to capture human activities in unlit areas (e.g., nighttime traffic) further supports data enhancement. Applying these enhanced data to SDG7 (sustainable energy) monitoring can also support assessments of energy access in off-grid regions.

When certain regions, such as those with weak TNL–EPC correlations, clearly show a tendency to deviate from the general pattern of other regions, separate analyses of these regions can be considered. Measures such as weighting the model parameters can be used to account for the uniqueness of these regions and mitigate the impact of provincial anomalies. Particularly, in regions with weak TNL–EPC correlations, exploring region-specific thresholding methods that account for unique local factors, such as industrial activities or agricultural burning, and the fusion of data from multiple sources can further improve model accuracy in different regions. These approaches can help improve model accuracy and generalisability by addressing regional specificities.

To optimise the model, the construction of separate models for industrial, residential, and commercial electricity use is considered necessary to avoid mixed signal interference (e.g., separation of high-tech zones and residential areas in Nanjing).

(4) The analysis of spatiotemporal variations in electricity consumption revealed that China’s electricity consumption has increased continuously over the past 12 years. The spatial distribution of high-consumption areas, centred around the Beijing–Tianjin–Hebei region, the Yangtze River Delta, the Pearl River Delta, provincial capitals, and sub-provincial cities on the eastern coast, has gradually expanded outward. This expansion is accompanied by increasing connectivity among these urban centres. Significant differences in electricity consumption are observed between the eastern and western regions, as well as between the coastal and interior areas, showing a strong correlation with topographic distribution.

Given the current situation, it is recommended to increase investment in distributed renewable energy in the central and eastern regions and enhance the utilisation of renewable power resources and the construction of power infrastructure in the western regions. High-consumption areas should accelerate power grid upgrades by prioritising the deployment of smart grids and distributed energy storage in hotspots such as the Yangtze River Delta and Pearl River Delta to boost the integration of new energy. Additionally, a regional early warning system for electricity consumption should be established, using spatialised data to monitor load fluctuations in real time.

(5) By ranking the increment and growth rates of electricity consumption of major cities in China from 2012 to 2023, we have learned about the development trajectories of major cities in China and found that the third- and fourth-tier cities in the south are poised to become significant contributors to future electricity consumption. This trend indicates a shift in economic activity and population growth toward these smaller cities, highlighting their emerging role in the national energy landscape.

To optimise power resource allocation and energy transition, targeted investments in emerging growth areas, including integrating third-tier cities in the South into the national grid expansion, are proposed in conjunction with the construction of new infrastructures such as photovoltaic rooftops and charging stations. Additionally, it is recommended to implement green transformation support for industrial cities, with specific subsidy policies for the old industrial bases in the Northeast, to promote the retrofitting of coal-fired units and industrial waste heat recovery, thereby reducing power demand fluctuations during the transition period.

(6) Finally, open data platforms and collaborative mechanisms are essential. The fragmented preprocessing methods for nighttime light data currently hinder the reproducibility and comparison of studies. Therefore, establishing an open-source data processing platform with modules for noise removal, data fusion and calibration, and supporting user-defined workflows (similar to Google Earth Engine), can promote interdisciplinary data sharing. Collaborating with energy departments to obtain detailed electricity consumption data (e.g., by industry and time of day) and building a “lighting-electricity” correlation database can also significantly enhance the depth and breadth of nighttime light data applications in the energy sector.

## Figures and Tables

**Table 1:** The five TNL–EPC models used in this work.

Regression Model	Regression Equation *
Linear	y = ax + b
Exponential	y = ae^bx^
Logarithmic	y = a + bln(x)
Power Function	y = ax^b^
Polynomial	y = k + a_1_x^1^ + a_2_x^2^ +…+a_n_x^n^

* Where x is the total nighttime light and y is the electricity consumption data; a and b are the coefficients; in the polynomial model, k is a constant term, a_1_, a_2_…a_i_, a_i+1_…a_n_ (i = 1,2…*n*) are the total nighttime light of the i order, and n is the highest order. To prevent overfitting, the value of n was taken as 2 in this paper.

**Table 2:** Provincial TNL–EPC model regression results, 2023. (n * = 27).

Regression Model	Regression Equation	R^2^
Power Function	y = 0.0005x^1.1424^	0.9584
Quadratic Polynomial	y = 2E−10x^2^ + 0.0035x − 242.28	0.9576
Linear	y = 0.004x − 489.07	0.9565
Exponential	y = 861.09e^1E−06x^	0.8649
Logarithmic	y = 3510.6ln(x) – 44,437	0.8361

* n denotes the number of samples involved in the model regression.

**Table 3:** Optimal regression results of the TNL–EPC model for different graded provinces in 2023.

Level	n	Optimal Regression Model	Regression Equation	R^2^
Grade 1	10	Power Function	y = 0.0005x^1.1404^	0.9600
Grade 2	10	Quadratic Polynomial	y = 2E−09x^2^ + 0.001x + 618.91	0.9227
Grade 3	7	Quadratic Polynomial	y = −2E−09x^2^ + 0.0075x − 1974.9	0.8440

**Table 4:** Provincial December 2020 TNL–EPC model regression results. (n = 27).

Regression Model	Regression Equation	R^2^
Quadratic Polynomial	y = 5E−12x^2^ + 0.0003x + 11.11	0.9381
Linear	y = 0.0003x + 6.9128	0.9380
Power Function	y = 7E−05x^1.1091^	0.9377
Logarithmic	y = 249.32ln(x) − 3065.3	0.8206
Exponential	y = 72.137e^1E−06x^	0.8057

**Table 5:** Year-by-year national TNL–EPC model regression results. (n = 12).

Regression Model	Regression Equation	R^2^
Quadratic Polynomial	y = 3E−11x^2^ + 0.0015x + 25,625	0.9706
Linear	y = 0.0026x + 14,490	0.9690
Exponential	y = 30,742e^4E−08x^	0.9687
Power Function	y = 0.1423x^0.7768^	0.9667
Logarithmic	y = 53,329ln(x) – 828,369	0.9519

**Table 6:** Comparison of the optimal regression results of the TNL–EPC model for ungraded and graded provinces in 2012 and 2023.

Year	Level	n	Regression Model	R^2^
2012	Non-hierarchical	27	Power Function	0.9257
	Grade 1	10	Quadratic Polynomial	0.9779
	Grade 2	10	Exponential	0.7763
	Grade 3	7	Quadratic Polynomial	0.7196
2023	Non-hierarchical	27	Power Function	0.9584
	Grade 1	10	Power Function	0.9600
	Grade 2	10	Quadratic Polynomial	0.9227
	Grade 3	7	Quadratic Polynomial	0.8440

**Table 7:** Comparison of the optimal regression results of the TNL–EPC model for ungraded and graded provinces in December 2020.

Level	n	Regression Model	R^2^
Non-hierarchical	27	Quadratic Polynomial	0.9381
Grade 1	10	Linear	0.9610
Grade 2	10	Quadratic Polynomial	0.7866
Grade 3	7	Linear	0.9477

**Table 8:** Comparison of the spatial and temporal regression results.

	Spatial Regression R^2^ *			Temporal Regression R^2^	
Annual Data	Ungraded		0.9584	0.9706	
	Graded	Grade 1	0.9600		
		Grade 2	0.9227		
		Grade 3	0.8440		
Monthly Data	Ungraded		0.9381		
	Graded	Grade 1	0.9610	National	0.3241
		Grade 2	0.7866	Provincial	0.8893
		Grade 3	0.9477	Prefectural	0.7427

* For the spatial regression, the annual data are for 2023 and the monthly data are for December 2020, for example.

**Table 9:** Ranking of China’s major prefectures, in terms of incremental and growth rates of electricity consumption in the past 12 years (top 10).

Incremental Ranking	Region	Increment (TWh)	Growth Rate Ranking	Region	Average Annual Growth Rate
1	Chongqing	74.22606	1	Sansha	42.40%
2	Wuhan	48.29887	2	Ledong	26.27%
3	Beijing	45.38285	3	Kokdara	22.14%
4	Hangzhou	43.98894	4	Tiemenguan	19.91%
5	Wenzhou	41.67780	5	Dongfang	18.51%
6	Ningbo	39.85519	6	Beitun	16.53%
7	Hefei	39.04451	7	Shanwei	15.27%
8	Yulin	37.35607	8	Yibin	15.16%
9	Baoding	36.96459	9	Chongzuo	14.95%
10	Suzhou	36.65714	10	Ganzhou	14.57%

**Table 10:** Ranking of China’s major prefectures in terms of incremental and growth rates of electricity consumption in the past 12 years (bottom 10).

Incremental Ranking	Region	Increment (TWh)	Growth Rate Ranking	Region	Average Annual Growth Rate
1	Daqing	−8.89857	1	Daqing	−2.27%
2	Fushun	−2.23233	2	Panzhihua	−2.24%
3	Benxi	−1.57516	3	Fushun	−2.15%
4	Panzhihua	−1.49476	4	Benxi	−2.12%
5	Liaoyang	−1.04039	5	Liaoyang	−0.87%
6	Wuzhishan	0.03753	6	Wuzhishan	0.86%
7	Shennongjia	0.05814	7	Yingkou	1.07%
8	Sansha	0.26951	8	Baotou	1.57%
9	Baoting	0.29448	9	Shanghai	1.72%
10	Qiongzhong	0.44190	10	Jilin	1.76%

## Data Availability

The NPP-VIIRS data used in this study are publicly available and can be downloaded from the NOAA website at https://eogdata.mines.edu/products/vnl/ (accessed on 27 December 2024). Due to privacy restrictions, China’s electricity consumption data requires an application for access. However, the aggregated results and processed spatialised products generated in this study can be obtained from the corresponding author. Researchers from academic institutions may apply for partial data access through the official portal of the National Energy Administration: http://www.nea.gov.cn (accessed on 25 August 2024).

## Appendix A

**Table A1:** Results of district grading in 2012 in three grades.

Level	Region
Grade 1	Shanghai, Tianjin, Beijing, Jiangsu, Zhejiang, Guangdong, Shandong, Liaoning, Fujian, Henan
Grade 2	Hebei, Anhui, Shanxi, Ningxia, Hainan, Shaanxi, Chongqing, Hubei, Jilin, Hunan
Grade 3	Guizhou, Guangxi, Sichuan, Jiangxi, Yunnan, Gansu, Qinghai

**Table A2:** Year-by-year provincial optimal TNL–EPC model regression results. (n = 12).

Region	R^2^	Region	R^2^	Region	R^2^
Chongqing	0.9898	Jiangxi	0.9623	Yunnan	0.9340
Zhejiang	0.9843	Ningxia	0.9574	Shandong	0.9247
Sichuan	0.9833	Guangxi	0.9549	Shanghai	0.9191
Anhui	0.9822	Liaoning	0.9547	Gansu	0.8591
Hebei	0.9784	Tianjin	0.9503	Guizhou	0.8533
Jiangsu	0.9760	Henan	0.9479	Jilin	0.8522
Hubei	0.9741	Hainan	0.9389	Xinjiang	0.7985
Fujian	0.9739	Beijing	0.9352	Heilongjiang	0.7485
Guangdong	0.9689	Hunan	0.9351	Tibet	0.2862
Shanxi	0.9674	Shaanxi	0.9349	Qinghai	0.2827

**Table A3:** Year-by-year prefectural optimal TNL–EPC model regression results. (n = 12).

Region	R^2^	Region	R^2^	Region	R^2^
Changsha	0.9921	Haikou	0.9559	Shenyang	0.9406
Hefei	0.9848	Wuhan	0.9532	Nanjing	0.9287
Nanchang	0.9810	Guangzhou	0.9471	Changchun	0.9249
Nanning	0.9684	Xian	0.9465	Chengdu	0.8897
Hangzhou	0.9618	Taiyuan	0.9457	Urumqi	0.8414
Jinan	0.9579	Zhengzhou	0.9431	Harbin	0.8224

**Table A4:** Month-by-month provincial optimal TNL–EPC model regression results. (n = 7).

Region	R^2^	Region	R^2^	Region	R^2^
Jilin	0.8893	Hainan	0.4126	Hunan	0.1389
Jiangsu	0.8837	Liaoning	0.357	Xinjiang	0.0754
Tianjin	0.8236	Qinghai	0.3287	Guangxi	0.0476
Heilongjiang	0.7616	Shandong	0.3268	Anhui	0.0411
Beijing	0.7551	Shaanxi	0.2984	Hubei	0.0323
Guizhou	0.7394	Gansu	0.2468	Sichuan	0.0260
Yunnan	0.5369	Henan	0.2266	Fujian	0.0222
Chongqing	0.4800	Shanxi	0.2007	Jiangxi	0.0129
Tibet	0.4615	Ningxia	0.2	Shanghai	0.0089
Hebei	0.4240	Guangdong	0.195	Zhejiang	0.0050

**Table A5:** Month-by-month prefectural optimal TNL–EPC model regression results. (n = 7).

Region	R^2^	Region	R^2^	Region	R^2^
Nanjing	0.8573	Taiyuan	0.6999	Zhengzhou	0.3160
Hohhot	0.7427	Shenyang	0.5719	Jinan	0.3142
Harbin	0.7282	Haikou	0.3490	Guiyang	0.0987

**Table A6:** Spatial dimension electricity consumption model, 2012–2023. (n = 27).

Year	Regression Model	R^2^	Year	Regression Model	R^2^
2012	y = 0.002x^1.052^	0.9257	2018	y = 0.0007x^1.1228^	0.9364
2013	y = 0.0024x^1.0375^	0.9274	2019	y = 0.0009x^1.1052^	0.9478
2014	y = 0.0032x^1.0206^	0.9416	2020	y = 0.0013x^1.0729^	0.9459
2015	y = 0.0015x^1.072^	0.9027	2021	y = 0.0011x^1.0817^	0.9516
2016	y = 0.0029x^1.0306^	0.9233	2022	y = 0.0007x^1.1124^	0.9529
2017	y = 0.0005x^1.1465^	0.9485	2023	y = 0.0005x^1.1424^	0.9584

**Table A7:** Results of the estimation of electricity consumption in the spatial dimension, 2012–2023. (n = 27).

Year	Actual Electricity Consumption (TWh)	Estimated Electricity Consumption (TWh)	Relative Error
2012	4563.371	4502.218954	−1.34%
2013	4882.357	4818.258709	−1.31%
2014	5042.500	4965.244957	−1.53%
2015	5131.880	4825.747606	−5.97%
2016	5384.275	5245.598265	−2.58%
2017	5720.500	5542.501405	−3.11%
2018	6191.400	6104.064916	−1.41%
2019	6466.000	6622.744086	2.42%
2020	6711.600	6551.464851	−2.37%
2021	7445.380	7110.030181	−4.50%
2022	7745.100	7533.999558	−2.73%
2023	8233.200	8557.112409	3.93%

**Table A8:** Estimated results of electricity consumption in the temporal dimension, 2012–2023. (n = 12).

Year	Actual Electricity Consumption (TWh)	Estimated Electricity Consumption (TWh)	Relative Error
2012	4965.653	5440.194119	9.56%
2013	5322.300	5661.476837	6.37%
2014	5521.314	5452.296386	−1.25%
2015	5549.957	5697.097546	2.65%
2016	5918.748	5483.017849	−7.36%
2017	6307.658	6769.662642	7.32%
2018	6844.909	7063.774151	3.20%
2019	7225.500	7280.501541	0.76%
2020	7511.000	7673.868468	2.17%
2021	8312.800	8523.437114	2.53%
2022	8637.200	8892.891354	2.96%
2023	9224.100	9717.996696	5.35%
